# Netrin-1 attenuates brain injury after middle cerebral artery occlusion via downregulation of astrocyte activation in mice

**DOI:** 10.1186/s12974-018-1291-5

**Published:** 2018-09-18

**Authors:** Xiaosong He, Yanqun Liu, Xiaohong Lin, Falei Yuan, Dahong Long, Zhijun Zhang, Yongting Wang, Aiguo Xuan, Guo-Yuan Yang

**Affiliations:** 1 Key Laboratory of Neuroscience, the Second Affiliated Hospital Guangzhou Medical University, Guangzhou, China; 2 Department of Anatomy, School of Basic Medical Science, Guangzhou Medical University, Guangzhou, China; 3 Department of Neurology, Changhai Hospital, Naval Military Medical University, Shanghai, China; 4 Hailisheng Biomarine Research Institute, Zhoushan, China; 5 Neuroscience and Neuroengineering Research Center, Med-X Research Institute and School of Biomedical Engineering, Shanghai Jiao Tong University, Shanghai, China; 6 Department of Neurology, Ruijin Hospital, School of Medicine, Shanghai Jiao Tong University, Shanghai, China; 7 Department of Anatomy, Guangzhou Medical college, Guangzhou, 511546 China; 8 Med-X Research Institute and School of Biomedical Engineering, 1954 Hua-shan Road, Shanghai, 200030 China

**Keywords:** Astrocyte, Brain, Ischemia, Inflammation, Netrin-1, UNC5H2

## Abstract

**Background:**

Netrin-1 functions largely via combined receptors and downstream effectors. Evidence has shown that astrocytes express netrin-1 receptors, including DCC and UNC5H2. However, whether netrin-1 influences the function of astrocytes was previously unknown.

**Methods:**

Lipopolysaccharide was used to stimulate the primary cultured astrocytes; interleukin release was used to track astrocyte activation. In vivo, shRNA and netrin-1 protein were injected in the mouse brain. Infarct volume, astrocyte activation, and interleukin release were used to observe the function of netrin-1 in neuroinflammation and brain injury after middle cerebral artery occlusion.

**Results:**

Our results demonstrated that netrin-1 reduced lipopolysaccharide-induced interleukin-1β and interleukin-12β release in cultured astrocytes, and blockade of the UNC5H2 receptor with an antibody reversed this effect. Additionally, netrin-1 increased p-AKT and PPAR-γ expression in primary cultured astrocytes. In vivo studies showed that knockdown of netrin-1 increased astrocyte activation in the mouse brain after middle cerebral artery occlusion (*p* < 0.05). Moreover, injection of netrin-1 attenuated GFAP expression (netrin-1 0.27 ± 0.06 vs. BSA 0.62 ± 0.04, *p* < 0.001) and the release of interleukins and reduced infarct volume after brain ischemia (netrin-1 0.27 ± 0.06 vs. BSA 0.62 ± 0.04 mm^3^, *p* < 0.05).

**Conclusion:**

Our results indicate that netrin-1 is an important molecule in regulating astrocyte activation and neuroinflammation in cerebral ischemia and provides a potential target for ischemic stroke therapy.

**Electronic supplementary material:**

The online version of this article (10.1186/s12974-018-1291-5) contains supplementary material, which is available to authorized users.

## Background

Stroke is a leading cause of death and disability worldwide. Currently, no effective drugs have shown benefit for patients in the acute phase of ischemic stroke except for tissue-type plasminogen activator (tPA). However, the short time window and the side effects limit the effective application of tPA. It is estimated that only 5% of ischemic stroke patients could benefit from tPA treatment [1]. Although numerous studies have shown that neuroprotection reduces brain injury after ischemic stroke in laboratory research, most neuroprotective attempts have failed when translated into the clinic. The lack of effective treatments for ischemic stroke has turned the efforts of researchers and physicians to the neuroprotective effects of not only neurons but also other cell types including astrocytes [2].

Astrocytes are the most abundant cells in the mammalian brain and display diverse functions including blood flow regulation, neurotransmitter metabolism, and endogenous neural stem cell development [3]. Under the cerebral ischemia condition, astrocytes are activated, which is characterized by an increase in glial fibrillary acidic protein (GFAP) expression and elongation of neural processes, as well as secretion of interleukins (ILs) [4]. Astrocytes play multiple roles in ischemic stroke pathology and can be a therapeutic target in promoting functional recovery after cerebral ischemia [5, 6]. Astrocytes respond differently to ischemic injury based on the distance from the lesion, on the severity, and on the phase course of ischemic stroke. In the acute phase, astrocytes produce inflammatory mediators such as ILs and chemokines, which initiate focal inflammatory and immune responses and aggravate brain injury after ischemia/reperfusion [7]. Meanwhile, the astrocytes adjacent to the lesion are activated and form a glial scar. In the first few days, the glial scar restricts the damage to remote areas [6]. However, glial scars have also been shown to inhibit the axon sprouting in the recovery phase of injury. In the chronic phase, studies demonstrated that activated astrocytes could secrete growth factors to aid the recovery [8]. Recently, it was demonstrated that activated astrocytes could form a glial scar, which may promote axon regeneration following severe spinal cord injury [9]. Despite the controversial role of astrocytes, it was widely accepted that astrocytes regulate neuroinflammation. Alleviating ischemia-induced astrocyte activation could promote recovery [10]. Hence, to explore the molecular mechanisms of astrocyte activation could assist the recovery post stroke.

The initiation function of netrin-1 (NT-1) in the brain is axon guidance and regulation of vascular development [11]. Recent studies have shown that NT-1 also exhibited an anti-inflammatory effect. For instance, NT-1 inhibited leukocytes migrating from the endothelium to the parenchyma in vitro and in vivo [12]. Exogenous NT-1 administration attenuated pulmonary inflammation during acute lung injury [13]. After brain ischemia, NT-1 improved recovery through enhanced remodeling ability and brain protection [14, 15]. However, whether NT-1 influences the neuroinflammation in the ischemic brain is unclear. The function of NT-1 mainly depends on the combined receptors such as Deleted in Colorectal Cancer (DCC) and UNC5H2. These receptors are expressed on astrocytes [16, 17]. However, the effect of NT-1 on astrocyte activation including the detailed molecular mechanism was previously unknown. Here, we explore the role of NT-1 in astrocyte activation and the neuroinflammation status after middle cerebral artery occlusion (MCAO) in mice.

## Methods

### Experimental design

Animal experiment procedure was approved by the Institutional Animal Care and Use Committee (IACUC), Guangzhou Medical University, China. Adult male CD-1 mice weighing 25 to 30 g were divided into five groups: (1) NT-1 injection group, mice underwent 2 μl NT-1 protein solution (100 ng/μl, dissolved in PBS containing 0.1% BSA) injection 1 day before MCAO; (2) BSA group, mice underwent BSA injection 1 day before MCAO; (3) NT-1 shRNA group, mice underwent lentivirus-carried NT-1shRNA injection 2 weeks before MCAO (shRNA sequence: GGGTGCCCTTCCAGTTCTA [18]); (4) GFP group, as control mice for the NT-1 shRNA group, these mice underwent GFP gene transfer through lentiviral vector injection; and (5) sham group, the mice underwent vessel occlusion without suture injection.

### Astrocyte culture

Primary astrocytes were prepared from C57BL/6 mice at the stage of P0 as previously described [8]. Astrocytes were cultured in six-well plates and were allowed to reach 90% confluence. Ten micrograms per milliliter of lipopolysaccharide was added in each well for 24 h to induce the interleukin release. NT-1 protein (500 ng/ml) and antibodies (UNC5H2, 5 μg/μl) were added into the medium 12 h before LPS intervention.

### Viral vector or NT-1 treatment

Adult CD-1 mice, weighted 25–28 g, were intraperitoneally anesthetized with pentobarbital (10 mg/kg body weight). The mouse skull was fixed on a stereotaxic plate (RWD, Shenzhen, China). A 0.5-mm bone hole was drilled by a hand drill (Fine Science Tool, Foster City, CA). Lentiviral vector (2.5 μl, 4 × 10^8^ IU/ml) or NT-1 was slowly injected into the left striatum (AP = − 0.02 mm, ML = − 2.5 mm, DV = 3 mm relative to the bregma) at the speed of 0.1 μl/min. After 20 min of injection, the needle was maintained in the brain for an additional 5 min. The bone hole was then sealed by bone wax and the skin was sutured. Animals were returned to their cages after being awaken. Two hundred nanograms of NT-1 protein (R&D system) was injected into the brain. NT-1 and BSA injection was performed 1 day before MCAO, lentiviral vectors carried NT-1 shRNA, or GFP gene was injected 2 weeks before MCAO.

### Mouse model of MCAO

The mouse model of MCAO was performed as previously reported [19]. Briefly, adult mice were anesthetized intraperitoneally with pentobarbital (10 mg/kg). Then, a 6-0 suture (Covidien, Mansfield, MA) with a round and silicone-coated tip was inserted from the left external carotid artery (ECA) into the internal carotid artery (ICA) to occlude the origin of middle cerebral artery (MCA). The animal body temperature was regularly maintained at 37 °C by a heating pad. After the surgery, animals were placed at room temperature and returned to the cage until awake. The CBF was measured at the MCA territory on the skull before surgery, after the occlusion and reperfusion respectively used a laser Doppler flowmetry (Moor instrument, Devon, UK). The CBF down to less than 10–15% after occlusion and return to more than 70% of baseline after reperfusion was recognized as the success of MCAO as shown in Table 1; no significance was observed among groups. In the sham group, mice underwent the same procedure as MCAO mice except suture injection. Three days after MCAO, mice were sacrificed and brain tissues were collected for further analysis.

**Table 1:** CBF measurement (Pu)

Groups	Before surgery	After occlusion	After reperfusion
NT-1 shRNA	277 ± 40	21 ± 6	270 ± 35
Scramble	266 ± 43	21 ± 6	233 ± 40
NT-1	265 ± 39	23 ± 4	230 ± 34
BSA	276 ± 34	25 ± 4	261 ± 35

### Immunohistochemistry

After animals were sacrificed, mice were perfused with saline and sequenced 4% paraformaldehyde fixation solution through the left ventricle and then quickly frozen in precooled isopentane. The brain was coronal sectioned to a 20-μm-thick slice. For immunofluorescence staining, sections were rinsed three times by PBS for 5 min, blocked by 5% normal donkey serum for 60 min, incubated with GFAP (1:300 dilution, Millipore Inc., Billerica, MA) and NT-1 (1:200 dilutions, Abcam, CA) antibodies at 4 °C overnight. After rinsing, sections were incubated with IgG for 60 min. For double immunofluorescence staining, sections were pretreated as described above; antibodies for DCC (1:100 dilutions, Santa Cruz, CA) and GFAP, A2B (1:200 dilutions, Santa Cruz) and GFAP, and UNC5H2 (1:100 dilutions, Abcam, Shanghai, China) and GFAP were incubated together; and corresponded secondary antibodies were incubated individually. The pictures were taken under confocal or fluorescent microscopes.

### Behavioral tests in mice

Modified neurologic severity scores (mNSS) of the animals were graded on a scale of 0 to 14, which is a composite of motor, reflex, and balance tests. The higher score reflected more severe injury. The elevated body swing test was performed as previously reported [20]. In this test, mice were held by the tail and raised 10 cm above the surface. The initial direction of swing was recorded in 20 trials in each mouse. The number of turns in each (left or right) direction was recorded for each mouse.

### Real-time PCR determination

Total RNA from astrocytes was isolated using TRIzol Reagent (Invitrogen). The RNA concentration was determined by a spectrophotometer (NanoDrop1000, Thermo, Wilmington, DE), followed by a reverse transcription process using PrimeScript RT reagent kit (TaKaRa, Dalian, China). RT-PCR amplification reactions were performed using SYBR Premix Ex Tag Kit according to the manufacturer by two steps under the following conditions: 95 °C for 30 s followed by 40 cycles of 95 °C for 5 s and 60 °C for 30 s. The primers used are listed in Table 2. The ΔCT method was used to detect the NT-1 receptors as described previously [21]. To quantify the mRNA levels of chemokines, the ΔΔCT method was used.

**Table 2:** Primers used for quantitative analysis of gene expression

Gene symbol	Forward primer 5′–3′	Reverse primer 5′–3′
DCC	CTCTTCACAGGATTGGAGAAAGGC	GAGGAGGTGTCCAACTCATGATG
UNC5H2	TGGATCTTTCAGCTCAAGACCCAG	AAGATGGCCAGCTGGAGCCG
A2B	TTGGCATTGGATTGACTC	TATGAGCAGTGGAGGAAG
IL-1β	GCAACTGTTCCTGAACTCAACT	ATCTTTTGGGGTCCGTCAACT
IL-12β	TGGTTTGCCATCGTTTTGCTG	ACAGGTGAGGTTCACTGTTTCT
GAPDH	AGGTCGGTGTGAACGGATTTG	TGTAGACCATGTAGTTGAGGTCA

### Western blot and ELISA analysis

After MCAO, mice were anesthetized by pentobarbital intraperitoneally as described above. Brains were quickly removed from a cooled brain mold and then cut into four coronal sections with 2 mm apart; the second section including ischemic core was collected. The section was divided into the cortex and striatum. For protein extraction, tissues were first homogenized by ultrasonic on ice and then centrifuged. Equal amount of total protein (40 μg) was loaded on 10% (*W*/*V*) SDS-PAGE and transferred to nitrocellulose membrane (Whatman, Piscataway, NJ). The membrane was blocked with 5% non-fat milk for 60 min at room temperature and subsequently with anti-NT-1 or anti-GFAP antibody (1:1000 dilutions, Abcam) and anti-β-actin antibody (1:1000 dilution, Santa Cruz) at 4 °C overnight. The membrane was washed in Tris-buffered saline Tween-20 (TBST) and incubated with appropriate horseradish peroxidase-conjugated anti-rabbit or anti-goat IgG for 120 min at room temperature and then reacted with an enhanced ECL substrate (Pierce, Rockford, IL). The result of chemiluminescence was recorded and semi-quantified by Quantity One imaging software (Bio-Rad, Hercules, CA).

The ELISA analysis was used to test the protein level of IL-1β and IL-12β in animals using ELISA kits (Neobioscience, China). Absorbance at 450 nm was recorded and the concentration of the target protein was quantified according to the standard curve. Result was expressed as picogram per milligram protein.

### Infarct volume measurement

For measuring brain infarct volume, a series of 20-μm-thick coronal sections with a 300-μm interval were cut. The sections were stained with 1% cresyl violet (Sigma) for 30 min at room temperature. Infarct volume was calculated by NIH Image J software. Infarct size equaled to the total contralateral hemisphere size minus total ipsilateral hemisphere size. Infarct volume between two adjacent sections was calculated by the formula: $V={\sum}_1^n\left[\left({S}_n+\sqrt{S_n\times {S}_{n+1}}+{S}_{n+1}\right)\times \frac{h}{3}\right]$, in which *h* was the distance between two adjacent sections and *S*_*n*_ and *S*_*n* + 1_ were the infarction areas of two adjacent sections. Total infarct volume was the sum of each adjacent section.

### Statistical analysis

Quantitative or semi-quantitative data are expressed as mean ± SD. ANOVA with Tukey multiple comparisons post-test was used for the analysis of multi groups. Comparisons between two groups were made by Student’s *t* test. A *p* < 0.05 was considered significantly different. Statistical analysis was carried out with Prism GraphPad 6 (GraphPad Software, Inc., La Jolla, CA).

## Results

### NT-1 attenuated the release of IL-1β and IL-12β in cultured astrocytes

**Fig. 1:**
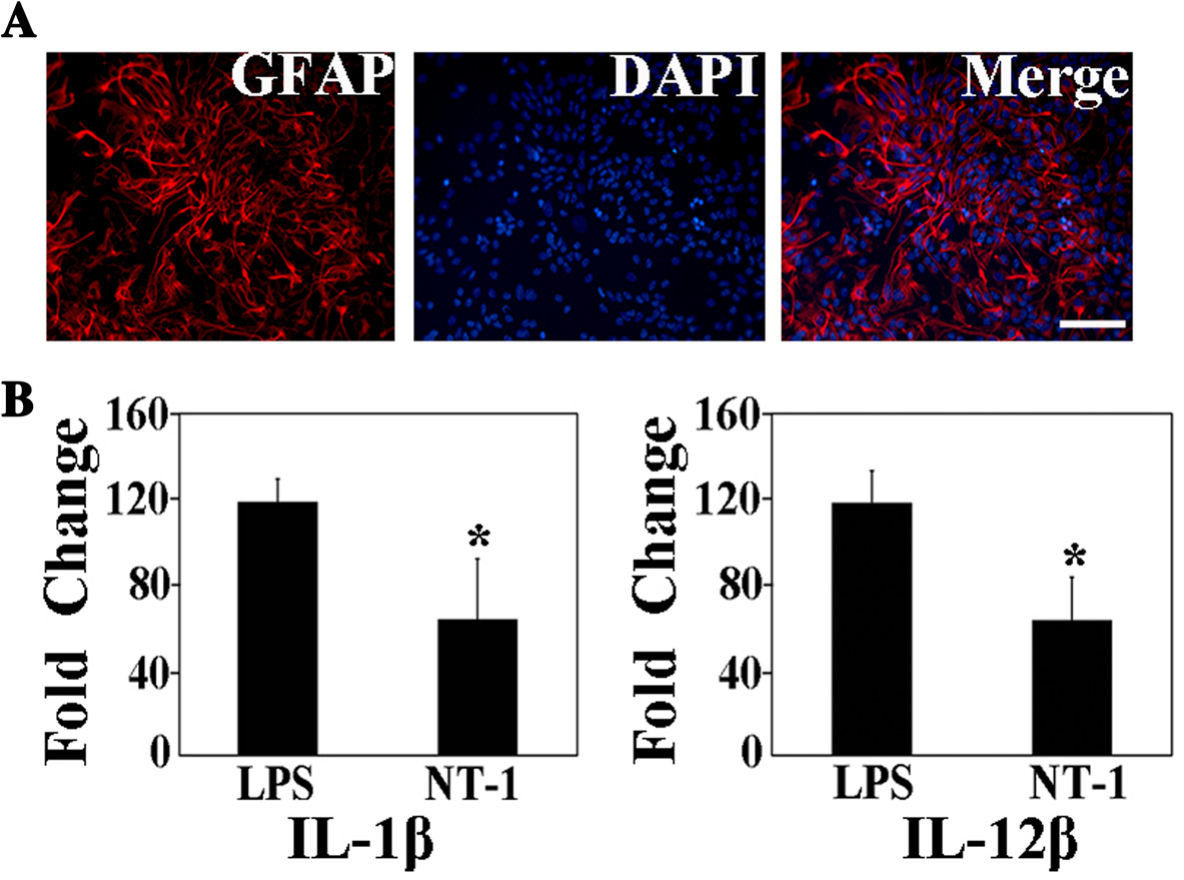
NT-1 inhibited interleukin release in primary cultured astrocytes. **a** The primary cultured astrocytes; the red indicates GFAP-positive cells, the blue represents the nucleus, and the merge shows the cultured GFAP-positive astrocytes; bar = 100 μm. **b** mRNA expression of the interleukin IL-1β and IL-12β in primary cultured astrocytes induced by LPS and NT-1 treatment; *p < 0.05 compared with the LPS group

Primary astrocytes were obtained from the cortex of P0 mice. Through counting the GFAP-positive cells, we determined that the purity of the cultured astrocytes was higher than 95% (Fig. 1a). No ionized calcium-binding adaptor molecule 1 (IBA1)-positive microglia were observed in the cultured cells (Additional file 1: Figure S1). To reveal the effects of NT-1 on astrocyte activation, primary astrocytes were stimulated by lipopolysaccharide (LPS). Although there are differences in gene expression and cellular mechanisms between LPS- and MCAO-induced astrocyte activation [22], LPS is widely used to stimulate astrocyte activation in ischemia condition [10]. In the present study, the release of IL-1β and IL-12β was used to track astrocyte activation. We found that LPS induced the release of IL-1β and IL-12β from cultured astrocytes, and NT-1 treatment before LPS intervention significantly attenuated IL-1β (118 ± 10.6 vs. 64 ± 28.3, *p* < 0.05) and IL-12β (118 ± 14.8 vs. 60 ± 20.1, *p* < 0.05) release, suggesting that NT-1 could be an important target in regulating astrocyte activation during neuroinflammation (Fig. 1b).

### UNC5H2 was the NT-1 receptor responsible for sustaining astrocyte activation

**Fig. 2:**
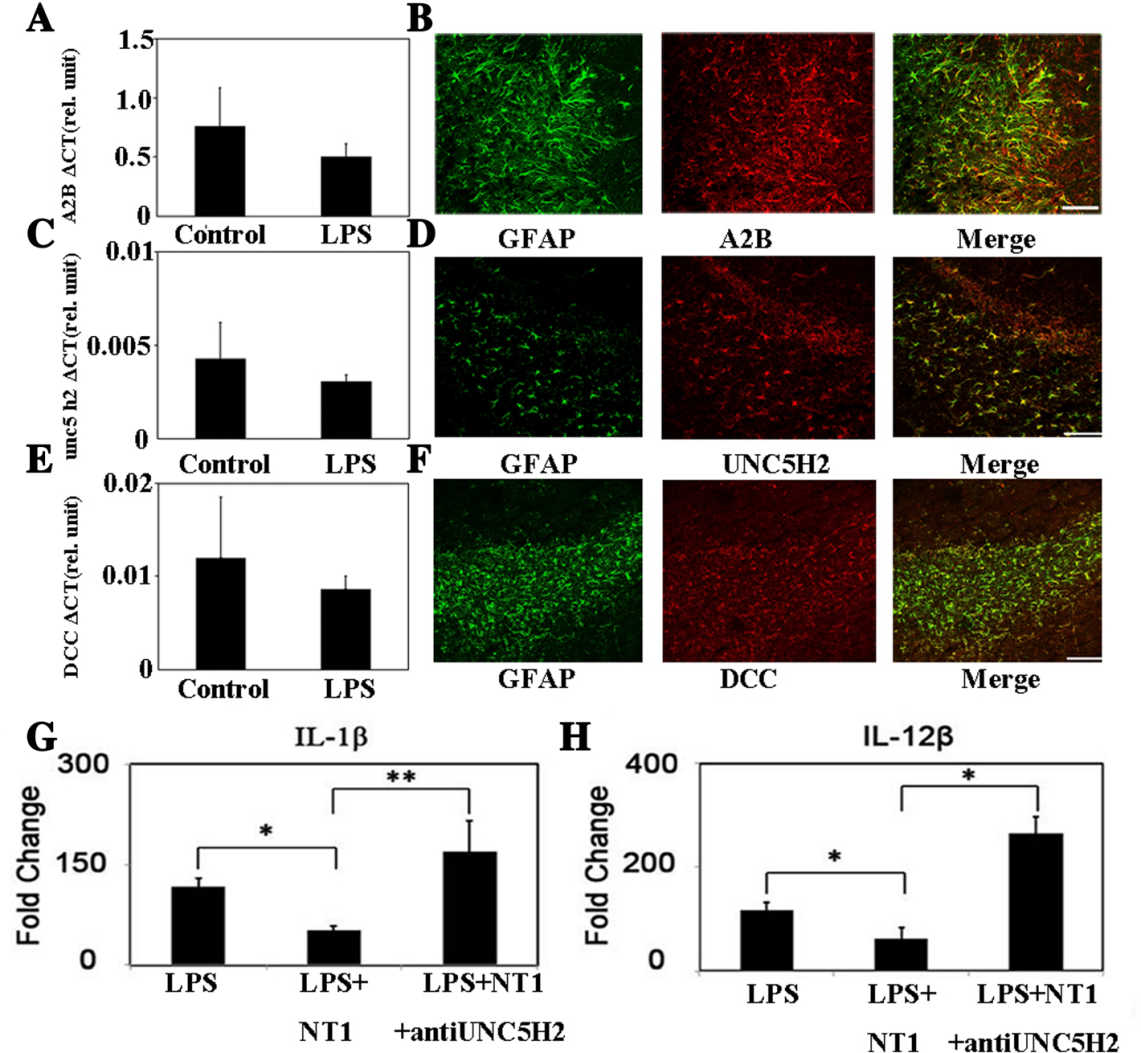
NT-1-induced inhibition of interleukin release in primary cultured astrocytes was dependent upon UNC5H2 receptors. **a** Bar graph exhibits the mRNA expression of A2B in the cultured astrocytes in different conditions. **b** The immunofluorescence results show A2B expression in the brain sections after ischemia; bar = 100 μm. **c** Bar graph exhibits the mRNA expression of UNC5H2 in the cultured astrocytes in different conditions. **d** The immunofluorescence results show UNC5H2 expression in the brain sections after ischemia; bar = 50 μm. **e** Bar graph exhibits the RNA expression of DCC in the cultured astrocytes in different conditions. **f** The immunofluorescence results show DCC expression in the brain sections after ischemia; bar = 100 μm. **g** IL-1β release in the astrocytes under different interventions; *p < 0.05, **p < 0.01. **h** IL-12β release in the astrocytes under different interventions; *p < 0.05, **p < 0.01

The effect of NT-1 depends on combined receptors [23]. Therefore, we tested NT-1 receptor expression. We found that the NT-1 receptors DCC, A2B, and UNC5H2 were expressed in the cultured astrocytes at the mRNA level (Fig. 2a, c, e). The immunostaining results demonstrated that DCC, A2B, and UNC5H2 were also present on astrocytes in the MCAO mouse brain tissue (Fig. 2b, d, f).

To define the receptors that are responsible for mediating the effect of NT-1 on astrocyte activation, we applied antibodies or an inhibitor in the cultured astrocytes to respectively neutralize or block the receptors before NT-1 intervention and LPS stimulation. The results showed that NT-1 pretreatment significantly attenuated IL-1β and IL-12β release, and administration of the UNC5H2 antibody in the culture medium reversed the inhibitory function of NT-1 (NT-1 + anti-UNC5H2 170 ± 46.6 vs. NT-1 51 ± 7.72, *p* < 0.05, Fig. 2g, h). In contrast, blocking NT-1 receptors by the DCC antibody or A2B receptor inhibitor did not alter IL-1β or IL-12β release compared to the control conditions. These results indicated that UNC5H2 was the key receptor in regulating astrocyte activation.

### NT-1 phosphorylated AKT and increased PPAR-γ expression

**Fig. 3:**
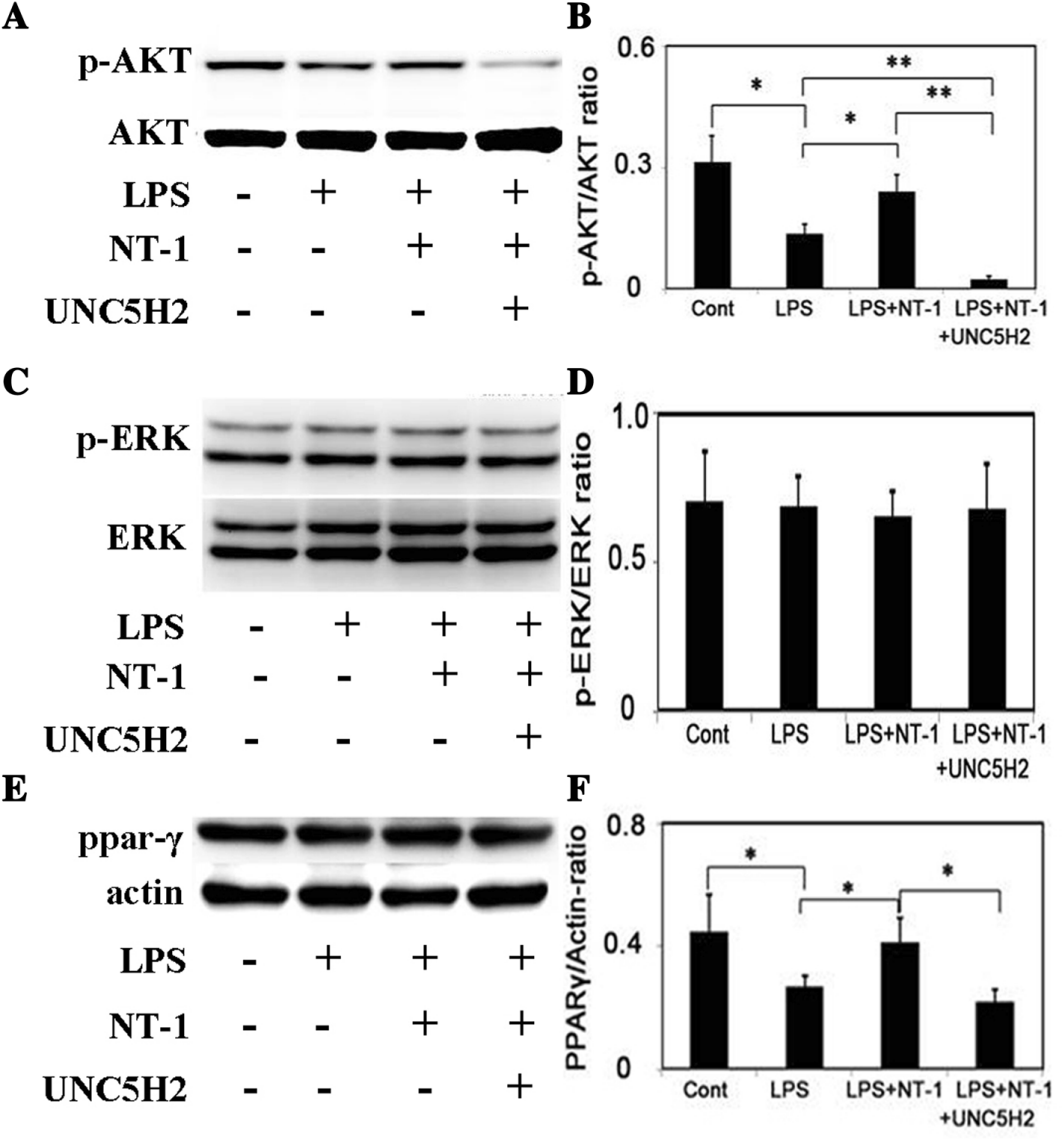
NT-1/UNC5H2 activated the AKT signaling pathway and PPAR-γ. **a** The expression of p-AKT and AKT analyzed by Western blot; the plus sign represents the material was added to the medium and the minus sign indicates the material was absent from the culture medium. **b** Bar graph represents the statistical results of p-AKT and AKT expression. **c** The expression of P-ERK and ERK examined by Western blot; the plus sign represents the material was added to the medium; the minus sign indicates the material was absent from the culture medium. **d** Bar graph represents the statistical results of p-AKT and AKT expression. **e** The expression of PPAR-γ and actin analyzed by Western blot; the plus sign represents the material was added to the medium; the minus sign indicates the material was absent from the culture medium. **f** Bar graph represents the statistical results of p-AKT and AKT expression; *p < 0.05, **p < 0.01

To identify the signaling pathways and the downstream molecular effectors involved in astrocyte activation, we performed Western blot to quantify phosphorylated extracellular signal-related kinase (p-ERK) and phosphorylated protein kinase B (p-AKT) expression in cultured astrocytes. Compared to the LPS-treated group, the group pretreated with NT-1 showed an increased p-AKT level in the primary astrocytes (*p* < 0.05, Fig. 3a, b) but not in p-ERK expression (Fig. 3c, d). In addition, Western blot results also showed that the expression of nuclear transcription factor peroxisome proliferator-activated receptor gamma (PPAR-γ) displayed a similar trend as p-AKT expression (*p* < 0.05, Fig. 3e, f). Furthermore, the UNC5H2 antibody attenuated p-AKT and PPAR-γ expression. These results suggest that the phosphoinositide 3-kinase (PI3K)/AKT signaling pathway is an important factor in mediating the function of NT-1 in astrocytes, and PPAR-γ is involved in this progress.

### NT-1 regulated astrocyte activation in an ischemic stroke animal model

Astrocyte activation is an important process that influences post-stroke recovery. To investigate whether NT-1 influences astrocyte activation after ischemic stroke, a lentivirus carrying NT-1 shRNA was injected into the mouse striatum to knock down NT-1 expression before the MCAO surgery and observe GFAP expression. To confirm the viral vector transduction in the brain, the viral vector-carried shRNA or GFP was injected into the peri-focal region (striatum). Consistent with our previous results [19], the GFP was majorly expressed in the striatum region 14 days after viral vector transduction (Additional file 2: Figure S2), where the peri-focal region is in our suture MCAO model.

**Fig. 4:**
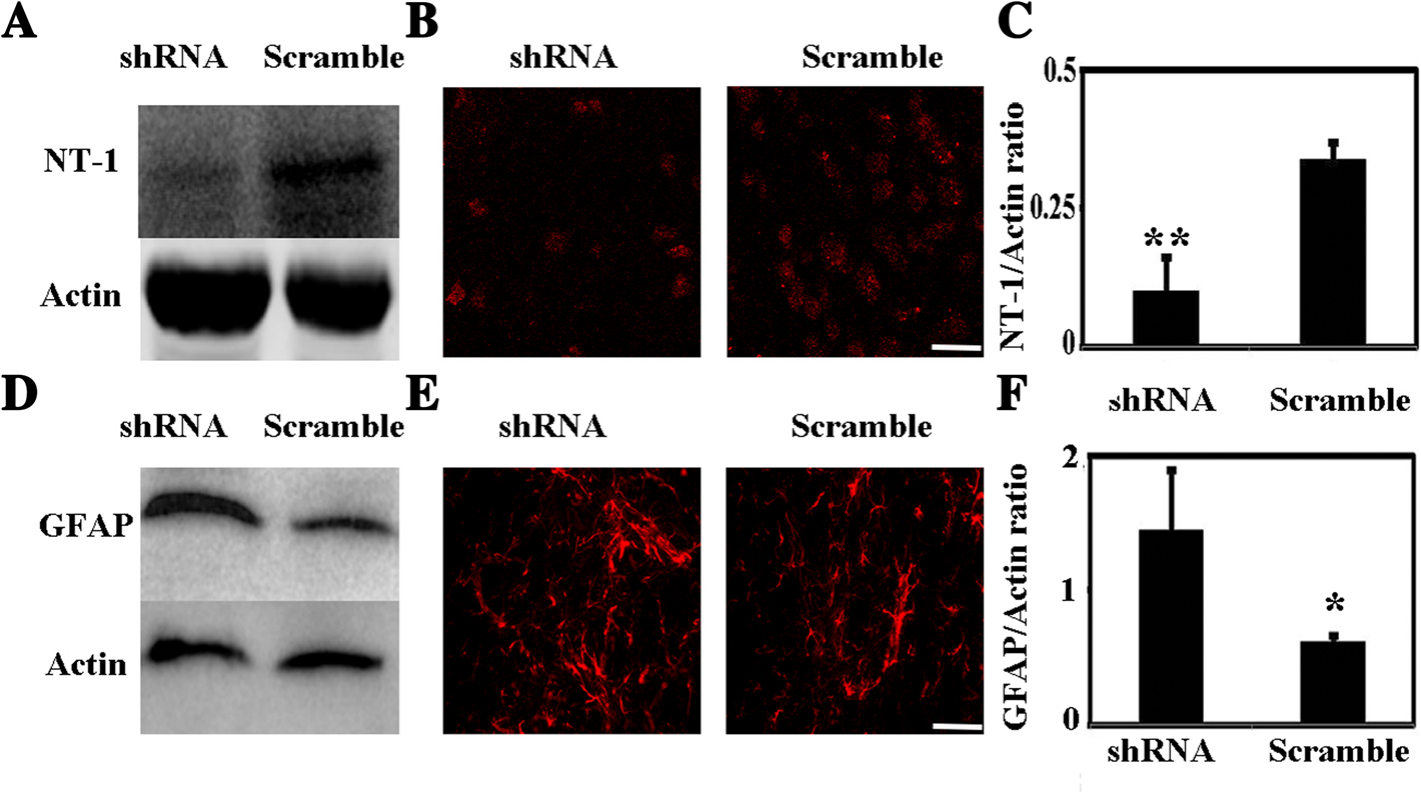
NT-1 knockdown promoted astrocyte activation in an animal model of MCAO. **a** NT-1 expression in the NT-1 shRNA-injected mice and the control group. **b** The immunofluorescence staining of NT-1 in the shRNA and control groups; bar = 30 μm. **c** The statistical results of NT-1 expression analyzed by Western blot; **p < 0.01. **d** GFAP expression in the NT-1 shRNA-injected mice and the control group after ischemic stroke. **e** The immunofluorescence results of GFAP expression in the shRNA and control groups; bar = 30 μm. **f** The statistical results of NT-1 expression examined by Western blot analysis; *p < 0.05, *n* = 3–5 in each group

Next, Western Blot was performed to detect NT-1 and GFAP protein expression. The results showed that the NT-1 protein level was significantly reduced in the mouse striatum 2 weeks after the NT-1 shRNA injection (*p* < 0.01, Fig. 4a, b, c). Furthermore, the NT-1 shRNA injection enhanced astrocyte activation, as indicated by GFAP expression (*p* < 0.01, Fig. 4d, e, f). These results suggest that NT-1 is an important regulator for astrocyte activation.

### NT-1 attenuated neuroinflammation and brain injury after MCAO

Inhibiting astrocyte activation could attenuate neuroinflammation and alleviate brain injury [24]. Given the role of NT-1 in astrocyte activation, we tested whether an NT-1 injection could alleviate neuroinflammation and reduce brain injury in an ischemic stroke animal model. NT-1 was injected in the mouse striatal area 1 day before MCAO surgery, and the neurological score and body swing test were used to assess behavioral performance 3 days after MCAO. To test the penetration of injected protein, Western blot analysis was performed to quantify NT-1 protein. The protein was extracted from the tissue surrounding the peri-focal region. Two hundred nanograms of recombinant mouse NT-1 was used as a positive control in Western blot tests. As shown in Additional file 3: Figure S3, NT-1 was increased in the brain 1 h after the NT-1 injection compared to the BSA-treated group, indicating the successful injection of protein.

**Fig. 5:**
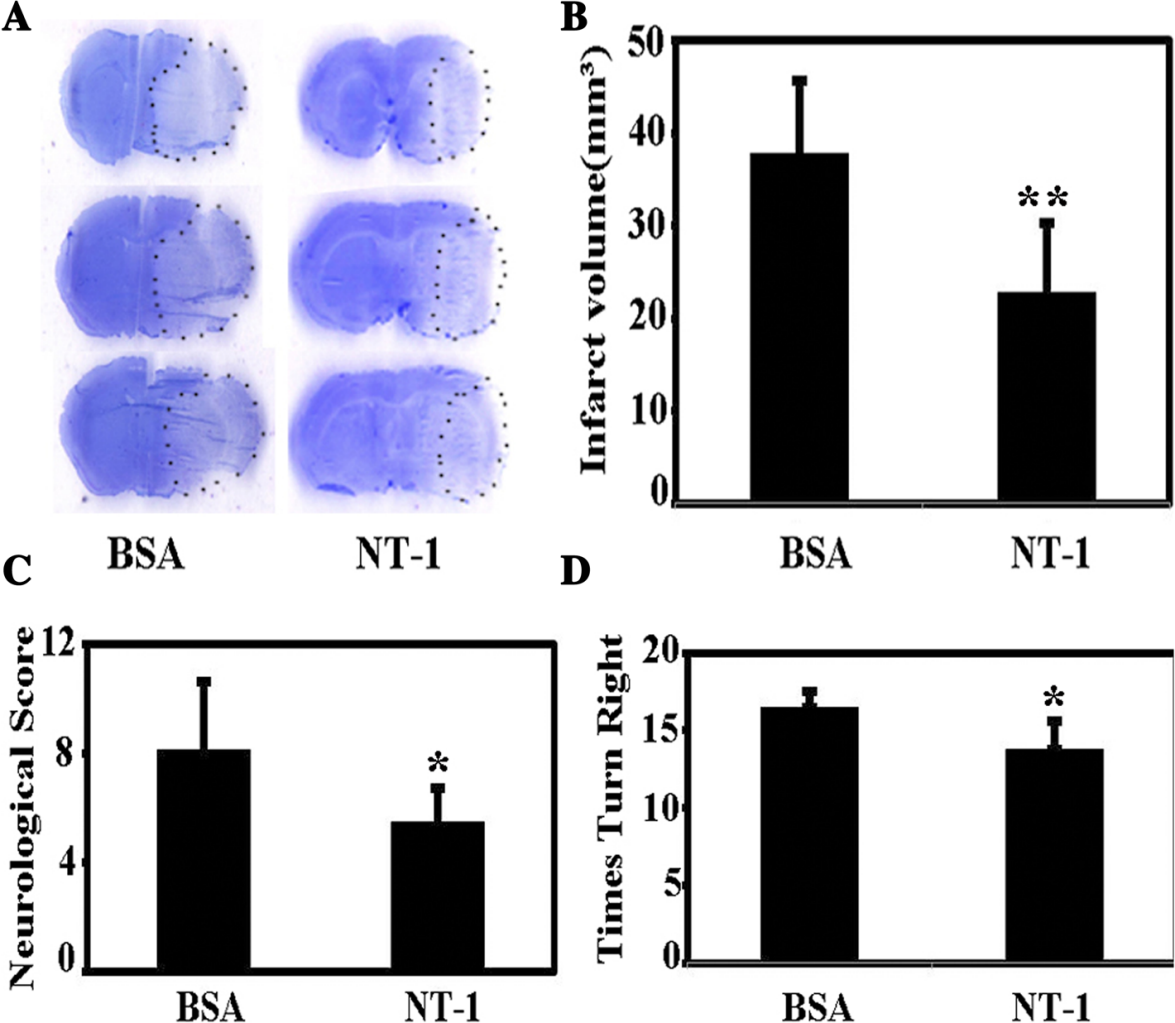
NT-1 alleviated brain injury and promoted recovery after MCAO in mice. **a** Crystal violet staining in the NT-1 protein- and BSA-injected groups 3 days after MCAO surgery. **b** The statistical results of infarct volume; **p < 0.01, *n* = 3 in each group. **c** The neurological scores of the two groups 3 days after MCAO surgery; *p < 0.05, *n* = 7 in each group. **d** The number of times the animals turned right in the body swing test; *p < 0.05, *n* = 7 in each group

**Fig. 6:**
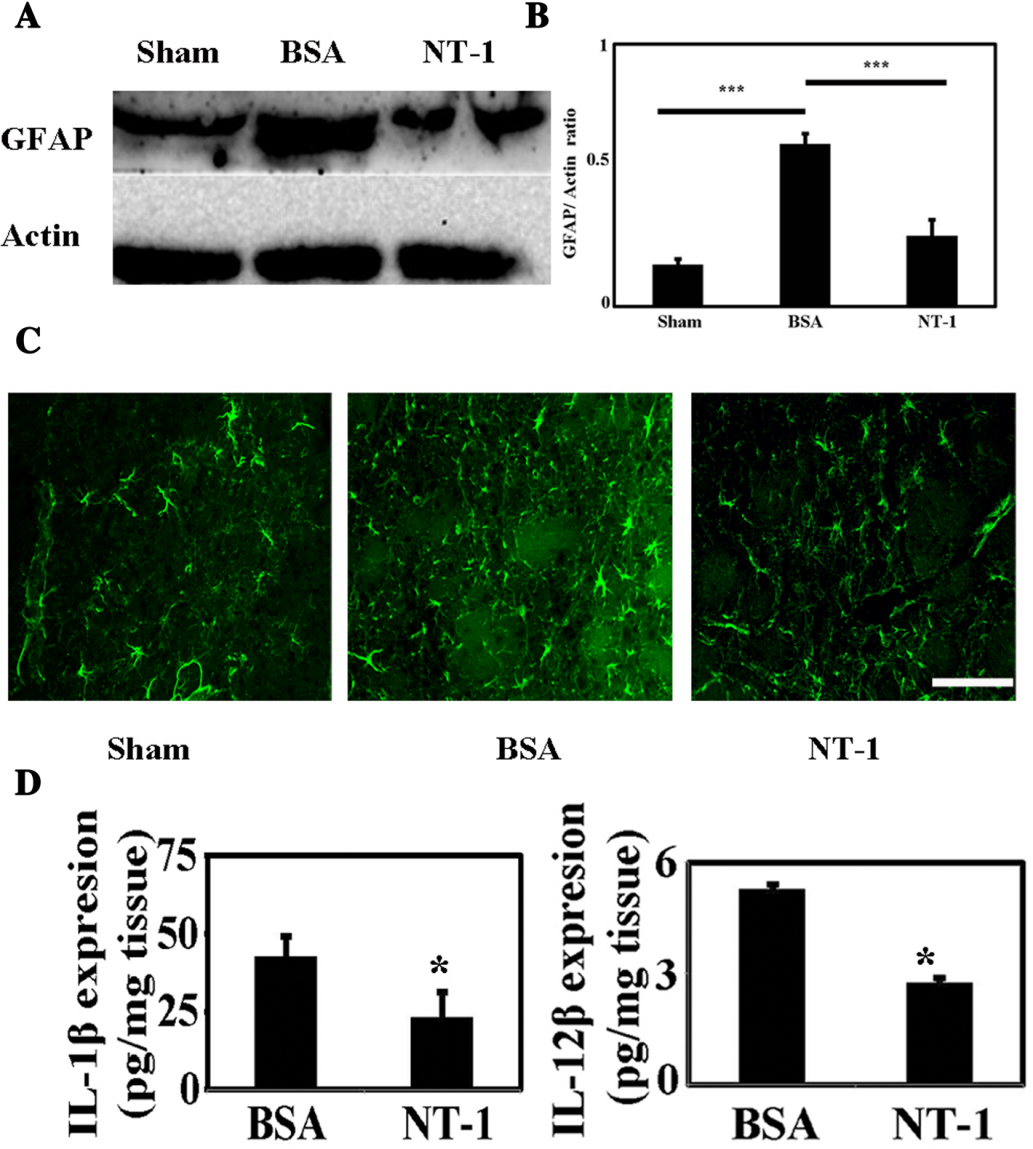
NT-1 reduced astrocyte activation and interleukin release after brain ischemia. **a** GFAP expression in the sham mice, NT-1 protein-injected mice, and the BSA-injected mice 3 days after MCAO. **b** The statistical results of GFAP expression in the NT-1 and control groups; ***p < 0.001. **c** The immunofluorescence results of GFAP staining in the peri-infarct area in the NT-1 and control groups; bar = 50 μm, *n* = 3 in each group. **d** ELISA results of the expression of chemokines IL-1β and IL-12β in the NT-1-injected mice and the control mice after ischemic stroke. The left graph indicates the IL-1β expression; the right graph indicates the IL-12β expression; *p < 0.05, *n* = 3–5 in each group

As shown in Fig. 5a, the infarct volume was significantly reduced in the NT-1 pretreatment group (NT-1 22.9 ± 7.5 vs. bovine serum albumin [BSA] 37.9 ± 7.8 mm^3^, *p* < 0.01), indicating that NT-1 alleviated brain injury 3 days after MCAO, and this was further shown by the neurological score (*p* < 0.05) and the number of times the animals turned to the right in the body swing test (*p* < 0.05, Fig. 5c, d). The Western blot results showed that the NT-1 injection attenuated GFAP expression (*p* < 0.05, Fig. 6a, b, c). The ELISA results indicated that injecting NT-1 attenuated chemokine IL-1β and IL-12β release (Fig. 6d). In addition, NT-1 has no influence on astrocyte activation or interleukin release in mice that did not undergo the MCAO surgery (Additional file 4: Figure S4). These results imply that NT-1 can attenuate neuroinflammation induced by ischemic stroke.

## Discussion

The effect of NT-1 in the brain depends on the combined receptors, the downstream signaling pathway, and the molecular effectors. Previous studies have demonstrated that DCC and UNC5H2 are expressed on astrocytes. However, the effect of NT-1 on astrocytes was not previously explored. In the present study, we demonstrated that pretreatment with NT-1 alleviated LPS-induced IL-1 and IL-12 release in cultured astrocytes. UNC5H2 was the receptor responsible for regulating the effect of NT-1 on astrocyte activation. Furthermore, NT-1 and UNC5H2 relied on the AKT pathway and PPAR-γ in regulating astrocyte activation. In the animal model, NT-1 knockdown via RNA silencing augmented the stroke-induced astrocyte activation, and NT-1 protein injection attenuated the ischemia-induced injury and subsequent neuroinflammation. In summary, we demonstrated a novel role of NT-1 in the prevention of ischemic stroke.

Recently, NT-1 was discovered to possess anti-inflammation potential in various disease models including acute lung injury and intestinal inflammation [25, 26]. Evidence also demonstrated that NT-1 was an important regulator in sustaining the integrity of the blood-brain barrier (BBB) during experimental autoimmune encephalomyelitis, traumatic brain injury, and ischemic stroke [21]. In the BBB, NT-1 was secreted by endothelial cells and provided anti-inflammation effects through decreasing chemokine secretion and protecting against cytokine-induced barrier disruption. Notably, NT-1 expression on endothelial cells was regulated by astrocyte-derived sonic hedgehog, which led us to hypothesize the effects of NT-1 on astrocytes. In this study, NT-1 protein was added to primary cultured astrocytes before LPS intervention, and we found that IL release was significantly reduced, indicating that NT-1 is an important factor in regulating the function of astrocytes. Furthermore, the in vivo results showed that NT-1 knockdown significantly increased GFAP expression, indicating that NT-1 is a key regulator in the process of astrocyte activation.

Receptors are the first factors that influence the function of NT-1. NT-1 has an array of receptors that are known to support the effect of NT-1, including DCC, UNC5H2, and A2B. In the present study, we found that three receptors could be detected in cultured astrocytes using real-time PCR and immunostaining in brain sections. Using an antibody or inhibitor blockade in vitro, we found that UNC5H2 was the receptor responsible for mediating the effect of NT-1 on astrocyte activation. UNC5H2 was also the prominent receptor while inducing the repelling function in guiding axon outgrowth and sustaining endothelial cell proliferation [27]. UNC5H2 was also required to inhibit leukocyte migration during inflammation and chemokine secretion and to induce the proliferation of Schwann cells [12, 28, 29]. Our results showed that the UNC5H2 antibody blockade significantly reversed the effects of NT-1 by inhibiting chemokine release from the cultured astrocytes. Interestingly, the concentration of the chemokines IL-1β and IL-12β released from astrocytes were even higher than that in the LPS group. Since antibody neutralizing was executed before NT-1 and LPS incubation, we believe that (1) UNC5H2 was the receptor responsible for NT-1-attenuated astrocyte activation and (2) NT-1 was not the only ligand of UNC5H2 in regulating astrocyte activation. In support of our postulation, using a knockout animal model, Yung et al. demonstrated that UNC5H2 was not the dominant receptor in mediating the action of NT-1 [30]. Of note, our data suggested that UNC5H2 was the receptor of NT-1 involved in regulating astrocyte activation.

NT-1 activates various signaling pathways and downstream effectors. For example, NT-1 interacted with DCC and activated the ERK pathway to promote the proliferation of endothelial cells and angiogenesis [31]. NT-1 combined with DCC to reduce the DNA damage and apoptosis in cultured primary neurons [32]. UNC5H2 was the receptor responsible for protecting BBB breakdown [33]. NT-1 also inhibited the notch signaling pathway after ischemic stroke [34]. In the process of angiogenesis and vascular remodeling, an NT-1/focal adhesion kinase (FAK)/Src signaling axis was also reported to be an important pathway [35]. In addition, NT-1 phosphorylated AMP-activated protein kinase (AMPK) and reduced the phosphorylation of mammalian target of rapamycin (mTOR) and P70S6K in a spinal cord injury model [36]. NT-1 also activated APPL1-AKT signing pathway via combining with DCC receptor to reduce apoptosis after subarachnoid hemorrhage in rats [37]. When introducing to astrocytes, AKT signing pathway was reported to protect astrocytes from activation [38]. In our study, we found a significant increase of *p*-AKT expression, while no change could be observed of ERK phosphorylation, when treated with NT-1 in astrocytes, indicating that NT-1 influenced the AKT pathway instead of the ERK pathways in inhibiting astrocyte activation.

Molecular effectors are crucial in regulating the effect of NT-1. When introduced to proliferating endothelial cells, NT-1 was reported to increase the expression of inducible nitric oxide synthase (iNOS) and cluster of differentiation (CD) 151 [31, 35]. NT-1 was also shown to be a regulator of nuclear factor kappa B (NF-κB) when inhibiting prostaglandin E2 (PGE-2) production [39]. NT-1 was also reported to be a key upstream regulator of PPAR-γ in reducing ischemia-induced inflammation [40]. PPAR-γ belongs to the nuclear hormone receptor family and was demonstrated to be an important target in the treatment of ischemic stroke. For example, PPAR-γ agonists were widely reported to attenuate ischemic brain injury through attenuating apoptosis and neuroinflammation [41]. PPAR-γ was also highly expressed on astrocytes and activation of PPAR-γ could protect astrocytes from apoptosis induced by ischemia [42]. Partly consistent with reported studies, we found that NT-1 significantly increased PPAR-γ expression in cultured astrocytes, and the UNC5H2 antibody blockade reversed the expression of PPAR-γ, indicating that PPAR-γ is an important effector in supporting the function of NT-1.

Numerous studies have demonstrated that NT-1 is an important factor influencing the recovery after ischemic stroke through various mechanisms [43]. For example, in the acute phase of brain ischemia, NT-1 attenuated apoptosis and promoted neural stem cell migration to alleviate brain injury [15]. It was also reported that NT-1 was able to enhance the local field potential (LFP) and reduce memory loss in a whole-brain ischemic model [44]. Recently, NT-1 was shown to attenuate injury through inhibiting autophagy [36]. In the chronic course of ischemic stroke, our previous results demonstrated that NT-1 was able to improve the remodeling process through enhanced angiogenesis, neurogenesis, and oligodendrogenesis [45–47]. Here, we report that NT-1 attenuates brain injury and reduces neuroinflammation via inhibiting astrocyte activation. Astrocyte activation has dual roles in post-stroke recovery depending on the upregulated genes. As reported, activated astrocytes can divide into the A1 and A2 types, where the A1 type loses much astrocyte-benefiting function and gains neurotoxic function, and the A2 type upregulates many neurotrophic factors [48]. Under ischemic conditions, astrocytes undergo apoptosis, which may increase the permeation of the BBB. However, the balance between apoptosis and proliferation sustains the astrocyte numbers [49]. In addition, it remains unknown which type of astrocytes could help to preserve the function of the BBB after ischemic stroke. Our results showed that NT-1 can inhibit GFAP expression, reduce chemokine release, and promote recovery, partly through reducing astrocytes activation, which expands the role of NT-1 in stroke pathology.

## Conclusion

In conclusion, our study demonstrated that NT-1 was a crucial factor in regulating astrocyte activation. The interaction of NT-1 with UNC5H2 was an important process in regulating astrocyte activation. NT-1 attenuated ischemia-induced inflammation through inhibiting astrocyte activation. Identification of this novel function of NT-1 has implications for research into the mechanisms of astrocyte activation and for the treatment of ischemic stroke.

## Additional files

Additional file 1: **Figure S1.** IBA1 staining in primary cultured cells. The red indicates IBA1-positive cells; the blue represents the nuclei; the merge shows the cultured IBA1-positive microglia. (JPG 179 kb)

Additional file 2: **Figure S2.** Virus expression in the brain. Lentivirus carried ShRNA, and GFP was majorly expressed in the striatum area 14 days after virus injection; green indicated GFP expression and blue represented Dapi. Bar = 500 μm. (JPG 394 kb)

Additional file 3: **Figure S3.** Protein penetration in the brain. NT-1 expression 1 h after injection; 200 ng recombinant mouse NT-1 was used as positive control. Actin was absent in the positive-control groups. (JPG 55 kb)

Additional file 4: **Figure S4.** NT-1 did not induce astrocyte activation in the intact mouse brain. a) GFAP expression in NT-1- and saline-treated mice that did not undergo MCAO. b) Cytokine IL-1β and IL-12β release in mice that did not undergo MCAO. (JPG 98 kb)

## Abbreviations

DCC: Deleted in Colorectal Cancer
GFAP: Glial fibrillary acidic protein
MCAO: Middle cerebral artery occlusion
NeuN: Neuronal nuclei
NT-1: Netrin-1
tPA: Tissue-type plasminogen activator
VEGF: Vascular endothelial growth factor

## References

[1] Campbell BC, Meretoja A, Donnan GA, Davis SM (2015). Twenty-year history of the evolution of stroke thrombolysis with intravenous alteplase to reduce long-term disability. Stroke.

[2] Barreto G, White RE, Ouyang Y, Xu L, Giffard RG (2011). Astrocytes: targets for neuroprotection in stroke. Cent Nerv Syst Agents Med Chem.

[3] Haydon P, Carmignoto G (2006). Astrocyte control of synaptic transmission and neurovascular coupling. Physiol Rev.

[4] Gleichman AJ, Carmichael ST (2013). Astrocytic therapies for neuronal repair in stroke. Neurosci Lett.

[5] Sims NR, Yew WP (2017). Reactive astrogliosis in stroke: contributions of astrocytes to recovery of neurological function. Neurochem Int.

[6] Liu Z, Chopp M (2015). Astrocytes, therapeutic targets for neuroprotection and neurorestoration in ischemic stroke. Prog Neurobiol.

[7] Li M, Li Z, Yao Y, Jin WN, Wood K, Liu Q, Shi FD, Hao J (2016). Astrocyte-derived interleukin-15 exacerbates ischemic brain injury via propagation of cellular immunity. Proc Natl Acad Sci U S A.

[8] Tang G, Liu Y, Zhang Z, Lu Y, Wang Y, Huang J, Li Y, Chen X, Gu X, Yang GY (2014). Mesenchymal stem cells maintain blood-brain barrier integrity by inhibiting aquaporin-4 upregulation after cerebral ischemia. Stem Cells.

[9] Anderson MA, Burda JE, Ren Y, Ao Y, O'Shea TM, Kawaguchi R, Coppola G, Khakh BS, Deming TJ, Sofroniew MV (2016). Astrocyte scar formation aids central nervous system axon regeneration. Nature.

[10] Liu T, Zhang T, Yu H, Shen H, Xia W (2014). Adjudin protects against cerebral ischemia reperfusion injury by inhibition of neuroinflammation and blood-brain barrier disruption. J Neuroinflammation.

[11] Bradford D, Cole SJ, Cooper HM (2009). Netrin-1: diversity in development. Int J Biochem Cell Biol.

[12] Ly NP, Komatsuzaki K, Fraser IP, Tseng AA, Prodhan P, Moore KJ, Kinane TB (2005). Netrin-1 inhibits leukocyte migration in vitro and in vivo. Proc Natl Acad Sci U S A.

[13] Mutz C, Mirakaj V, Vagts DA, Westermann P, Waibler K, Konig K, Iber T, Noldge-Schomburg G, Rosenberger P (2010). The neuronal guidance protein netrin-1 reduces alveolar inflammation in a porcine model of acute lung injury. Crit Care.

[14] Sun Hui, Le Thang, Chang Tiffany T.J., Habib Aisha, Wu Steven, Shen Fanxia, Young William L., Su Hua, Liu Jialing (2011). AAV-mediated netrin-1 overexpression increases peri-infarct blood vessel density and improves motor function recovery after experimental stroke. Neurobiology of Disease.

[15] Wu TW, Li WW, Li H (2008). Netrin-1 attenuates ischemic stroke-induced apoptosis. Neuroscience.

[16] Wang X, Xu J, Gong J, Shen H (2013). Expression of netrin-1 and its receptors, deleted in colorectal cancer and uncoordinated locomotion-5 homolog B, in rat brain following focal cerebral ischemia reperfusion injury. Neural Regen Res.

[17] Liu N, Huang H, Lin F, Chen A, Zhang Y, Chen R, Du H (2011). Effects of treadmill exercise on the expression of netrin-1 and its receptors in rat brain after cerebral ischemia. Neuroscience.

[18] Xu H, Liu J, Xiong S, Le YZ, Xia X (2012). Suppression of retinal neovascularization by lentivirus-mediated netrin-1 small hairpin RNA. Ophthalmic Res.

[19] He X, Lu Y, Lin X, Jiang L, Tang Y, Tang G, Chen X, Zhang Z, Wang Y, Yang GY (2017). Optical inhibition of striatal neurons promotes focal neurogenesis and neurobehavioral recovery in mice after middle cerebral artery occlusion. J Cereb Blood Flow Metab.

[20] Li J, Tang Y, Wang Y, Tang R, Jiang W, Yang GY, Gao WQ (2014). Neurovascular recovery via co-transplanted neural and vascular progenitors leads to improved functional restoration after ischemic stroke in rats. Stem Cell Reports.

[21] Podjaski C, Alvarez JI, Bourbonniere L, Larouche S, Terouz S, Bin JM, Lecuyer MA, Saint-Laurent O, Larochelle C, Darlington PJ (2015). Netrin 1 regulates blood-brain barrier function and neuroinflammation. Brain.

[22] Zamanian JL, Xu L, Foo LC, Nouri N, Zhou L, Giffard RG, Barres BA (2012). Genomic analysis of reactive astrogliosis. J Neurosci.

[23] Xu K, Wu Z, Renier N, Antipenko A, Tzvetkova-Robev D, Xu Y, Minchenko M, Nardi-Dei V, Rajashankar KR, Himanen J (2014). Neural migration. Structures of netrin-1 bound to two receptors provide insight into its axon guidance mechanism. Science.

[24] Shao W, Zhang SZ, Tang M, Zhang XH, Zhou Z, Yin YQ, Zhou QB, Huang YY, Liu YJ, Wawrousek E (2012). Suppression of neuroinflammation by astrocytic dopamine D2 receptors via alphaB-crystallin. Nature.

[25] He J, Zhao Y, Deng W, Wang DX (2014). Netrin-1 promotes epithelial sodium channel-mediated alveolar fluid clearance via activation of the adenosine 2B receptor in lipopolysaccharide-induced acute lung injury. Respiration.

[26] Aherne CM, Collins CB, Eltzschig HK (2014). Netrin-1 guides inflammatory cell migration to control mucosal immune responses during intestinal inflammation. Tissue Barriers.

[27] Larrivee B, Freitas C, Trombe M, Lv X, Delafarge B, Yuan L, Bouvree K, Breant C, Del Toro R, Brechot N (2007). Activation of the UNC5B receptor by Netrin-1 inhibits sprouting angiogenesis. Genes Dev.

[28] Tadagavadi RK, Wang W, Ramesh G (2010). Netrin-1 regulates Th1/Th2/Th17 cytokine production and inflammation through UNC5B receptor and protects kidney against ischemia–reperfusion injury. J Immunol.

[29] Lee HK, Seo I, Seo E, Seo SY, Lee HJ, Park HT (2007). Netrin-1 induces proliferation of Schwann cells through Unc5b receptor. Biochem Biophys Res Commun.

[30] Yung AR, Nishitani AM, Goodrich LV (2015). Phenotypic analysis of mice completely lacking netrin 1. Development.

[31] Nguyen A, Cai H (2006). Netrin-1 induces angiogenesis via a DCC-dependent ERK1/2-eNOS feed-forward mechanism. Proc Natl Acad Sci.

[32] Chen J, Du H, Zhang Y, Chen H, Zheng M, Lin P, Lan Q, Yuan Q, Lai Y, Pan X (2017). Netrin-1 prevents rat primary cortical neurons from apoptosis via the DCC/ERK pathway. Front Cell Neurosci.

[33] Yu J, Li C, Ding Q, Que J, Liu K, Wang H, Liao S (2017). Netrin-1 ameliorates blood-brain barrier impairment secondary to ischemic stroke via the activation of PI3K pathway. Front Neurosci.

[34] Yang X, Li S, Li B, Wang X, Sun C, Qin H, Sun H (2017). Netrin-1 overexpression improves neurobehavioral outcomes and reduces infarct size via inhibition of the notch1 pathway following experimental stroke. J Neurosci Res.

[35] Yang X, Li S, Zhong J, Zhang W, Hua X, Li B, Sun H (2016). CD151 mediates netrin-1-induced angiogenesis through the Src-FAK-Paxillin pathway. J Cell Mol Med.

[36] Bai L, Mei X, Shen Z, Bi Y, Yuan Y, Guo Z, Wang H, Zhao H, Zhou Z, Wang C (2017). Netrin-1 improves functional recovery through autophagy regulation by activating the AMPK/mTOR signaling pathway in rats with spinal cord injury. Sci Rep.

[37] Xie Z, Huang L, Enkhjargal B, Reis C, Wan W, Tang J, Cheng Y, Zhang JH (2017). Intranasal administration of recombinant Netrin-1 attenuates neuronal apoptosis by activating DCC/APPL-1/AKT signaling pathway after subarachnoid hemorrhage in rats. Neuropharmacology.

[38] Sankar Sitara B., Donegan Rebecca K., Shah Kajol J., Reddi Amit R., Wood Levi B. (2018). Heme and hemoglobin suppress amyloid β–mediated inflammatory activation of mouse astrocytes. Journal of Biological Chemistry.

[39] Ranganathan PV, Jayakumar C, Mohamed R, Dong Z, Ramesh G (2013). Netrin-1 regulates the inflammatory response of neutrophils and macrophages, and suppresses ischemic acute kidney injury by inhibiting COX-2-mediated PGE2 production. Kidney Int.

[40] Ranganathan PV, Jayakumar C, Ramesh G (2013). Netrin-1-treated macrophages protect the kidney against ischemia-reperfusion injury and suppress inflammation by inducing M2 polarization. Am J Physiol Renal Physiol.

[41] Cuartero MI, Ballesteros I, Moraga A, Nombela F, Vivancos J, Hamilton JA, Corbí ÁL, Lizasoain I, Moro MA (2013). N2 neutrophils, novel players in brain inflammation after stroke: modulation by the PPARγ agonist rosiglitazone. Stroke.

[42] Xia P, Pan Y, Zhang F, Wang N, Wang E, Guo Q, Ye Z (2018). Pioglitazone confers neuroprotection against ischemia-induced pyroptosis due to its inhibitory effects on HMGB-1/RAGE and Rac1/ROS pathway by activating PPAR. Cell Physiol Biochem.

[43] Tsuchiya A, Hayashi T, Deguchi K, Sehara Y, Yamashita T, Zhang H, Lukic V, Nagai M, Kamiya T, Abe K (2007). Expression of netrin-1 and its receptors DCC and neogenin in rat brain after ischemia. Brain Res.

[44] Bayat M, Baluchnejadmojarad T, Roghani M, Goshadrou F, Ronaghi A, Mehdizadeh M (2012). Netrin-1 improves spatial memory and synaptic plasticity impairment following global ischemia in the rat. Brain Res.

[45] Lu H, Wang Y, He X, Yuan F, Lin X, Xie B, Tang G, Huang J, Tang Y, Jin K (2012). Netrin-1 hyperexpression in mouse brain promotes angiogenesis and long-term neurological recovery after transient focal ischemia. Stroke.

[46] Lu H, Song X, Wang F, Wang G, Wu Y, Wang Q, Wang Y, Yang GY, Zhang Z (2016). Hyperexpressed netrin-1 promoted neural stem cells migration in mice after focal cerebral ischemia. Front Cell Neurosci.

[47] He X, Li Y, Lu H, Zhang Z, Wang Y, Yang GY (2013). Netrin-1 overexpression promotes white matter repairing and remodeling after focal cerebral ischemia in mice. J Cereb Blood Flow Metab.

[48] Liddelow SA, Guttenplan KA, Clarke LE, Bennett FC, Bohlen CJ, Schirmer L, Bennett ML, Munch AE, Chung WS, Peterson TC (2017). Neurotoxic reactive astrocytes are induced by activated microglia. Nature.

[49] Barreto GE, Sun X, Xu L, Giffard RG (2011). Astrocyte proliferation following stroke in the mouse depends on distance from the infarct. PLoS One.

