# Supplementary figures and images for: Netrin-1 attenuates brain injury after middle cerebral artery occlusion via downregulation of astrocyte activation in mice
Source: J Neuroinflammation. 2018 Sep 18;15:268. doi: 10.1186/s12974-018-1291-5 (PMC6145326; doi:10.1186/s12974-018-1291-5)

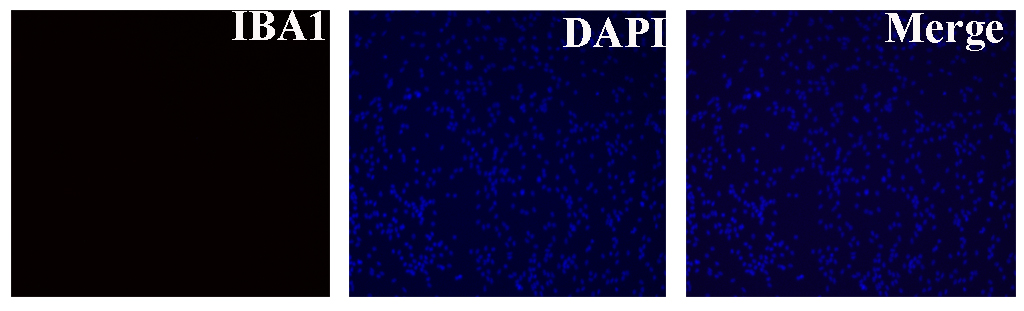

Supplement: Supplementary file 1 — Figure S1. IBA1 staining in primary cultured cells. The red indicates IBA1-positive cells; the blue represents the nuclei; the merge shows the cultured IBA1-positive microglia. (JPG 179 kb) [file 12974_2018_1291_MOESM1_ESM.jpg]

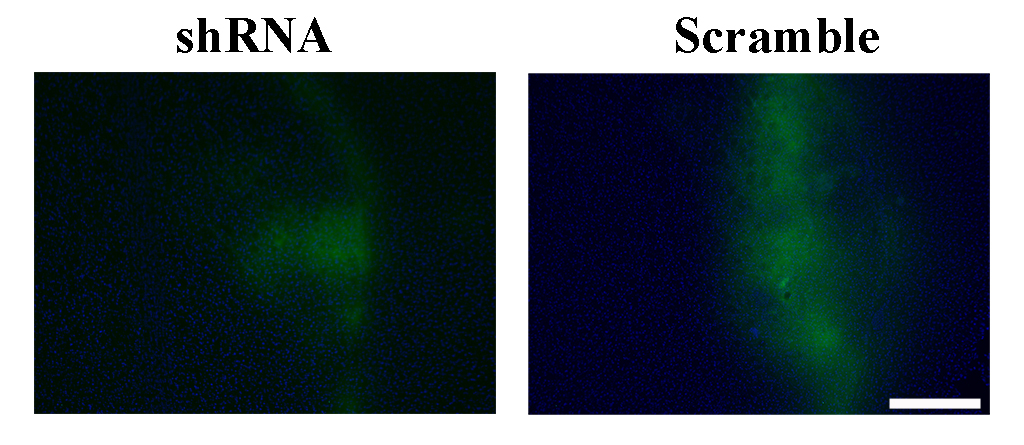

Supplement: Supplementary file 2 — Figure S2. Virus expression in the brain. Lentivirus carried ShRNA, and GFP was majorly expressed in the striatum area 14 days after virus injection; green indicated GFP expression and blue represented Dapi. Bar = 500 μm. (JPG 394 kb) [file 12974_2018_1291_MOESM2_ESM.jpg]

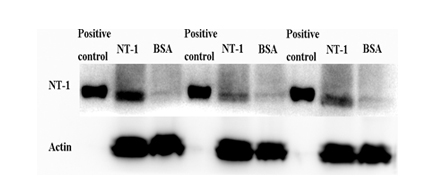

Supplement: Supplementary file 3 — Figure S3. Protein penetration in the brain. NT-1 expression 1 h after injection; 200 ng recombinant mouse NT-1 was used as positive control. Actin was absent in the positive-control groups. (JPG 55 kb) [file 12974_2018_1291_MOESM3_ESM.jpg]

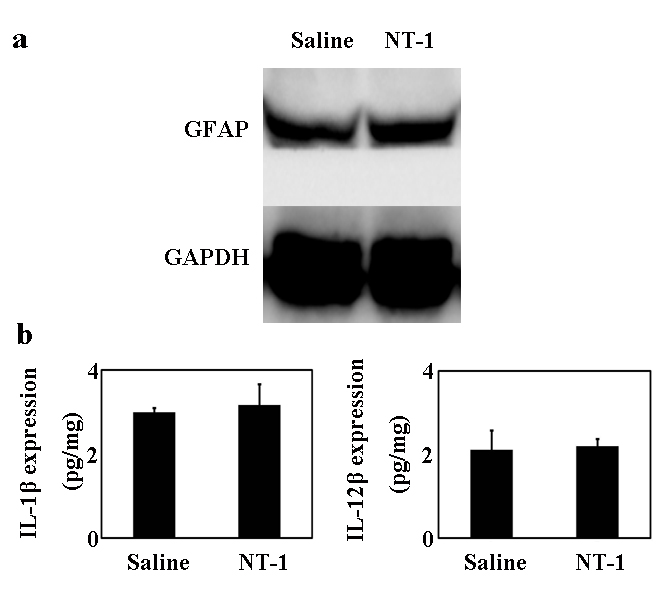

Supplement: Supplementary file 4 — Figure S4. NT-1 did not induce astrocyte activation in the intact mouse brain. a) GFAP expression in NT-1- and saline-treated mice that did not undergo MCAO. b) Cytokine IL-1β and IL-12β release in mice that did not undergo MCAO. (JPG 98 kb) [file 12974_2018_1291_MOESM4_ESM.jpg]
